# Integrated Control Strategies for a Precision Long-Travel Stage: Applications in Micro-Lens Fabrication

**DOI:** 10.3390/mi16101105

**Published:** 2025-09-28

**Authors:** Fu-Cheng Wang, Yan-Teng Chang, Ming-Hsiang Chang, Bo-Xuan Zhong, Tien-Tung Chung, Jia-Yush Yen

**Affiliations:** 1 Department of Mechanical Engineering, National Taiwan University, Taipei 10617, Taiwan; mazda5113@gmail.com (Y.-T.C.); ctas210018@gmail.com (M.-H.C.); a27845361@gmail.com (B.-X.Z.); ttchung@ntu.edu.tw (T.-T.C.); or jyen@mail.ntust.edu.tw (J.-Y.Y.); 2 Department of Mechanical Engineering, National Taiwan University of Science and Technology, Taipei 10617, Taiwan

**Keywords:** precision stage, model estimation, control switching, gain scheduling, feedforward, robust control, two-photon polymerization, micro-lens

## Abstract

This paper develops multiple control strategies for a precision long-travel stage, which comprises motor and piezoelectric transducer (PZT) stages. First, the PZT stage is equipped with control switching and model estimation mechanisms to achieve nm-level precision within 100 μm distances. The control switching mechanism selects the optimal control sequences by predicting system responses, while the model estimation algorithm updates the system model to improve the prediction accuracy. Second, the motor stage is equipped with gain-scheduling and feedforward control mechanisms to achieve a maximum displacement of 100 mm with a resolution of 0.1 μm. The gain scheduling control modifies the control gain in accordance with tracking errors, while the feedforward control can mitigate phase lags. We integrate the stages to achieve nm-level precision over long travels and conduct simulations and experiments to show the advantages of the control mechanisms. Finally, we apply the long-travel precision stage to fabricate micro-lenses using two-photon polymerization and evaluate the fabricated micro-lenses’ optical characteristics to illustrate the merits of the control strategies.

## 1. Introduction

The progression of technological advancements has critically increased the demand for high-precision positioning control. Precision engineering now extensively utilizes piezoelectric materials due to their advantageous properties, including rapid response times and high energy efficiency. Nevertheless, the inherent nonlinearities of these materials can significantly impair positioning accuracy. To address these challenges, researchers have developed various nonlinear modeling techniques and control strategies. For example, Chen et al. [1] proposed a hysteresis modeling approach that captured the nonlinear behaviors of piezoelectric actuators. Baziyad et al. [2] introduced an inverse Preisach model and integrated it with support vector machines to compensate for nonlinearities in piezoelectric-driven positioning systems. Keshavarzpour and Ghasemi [3] implemented a hybrid fuzzy proportional-integral-derivative (PID) control to suppress sound radiation and enhance disturbance rejection. McCartney et al. [4] proposed a compact model for a piezoelectric transistor to simulate nanoscale electromechanical behavior. However, these design approaches have not been entirely successful in eliminating the nonlinearities associated with piezoelectric materials.

Control system design typically involves balancing multiple, and often conflicting, performance criteria. For example, some controllers provide rapid responses but may result in significant overshoots, whereas others provide smoother responses but at the cost of prolonged settling times. To overcome the limitations of fixed controllers, numerous studies have investigated the use of switching control strategies that can provide dynamic adaptation to system conditions. For instance, Kim et al. [5] developed a mode-switching control scheme that integrated optimal servomechanisms and time-delay control to improve positioning accuracy and reduce transition errors in manufacturing equipment. Bashash et al. [6] implemented a switched-control mechanism that transited between two controllers according to position errors to fabricate a piezoelectric transducer (PZT) stage. Li et al. [7] combined trajectory planning and nonlinear feedback to develop a switching mode control for motor systems. Artetxe et al. [8] built a combined control using feedforward neural networks and sliding mode control to suppress hysteresis and improve tracking accuracy. However, these approaches did not address all the issues hindering the control of precision stages.

An integral control method for precision stages, proposed in [9], eliminated steady-state errors and outperformed fixed-gain controllers through adaptive adjustment of the control gain according to position errors. Zhang and Yan [10] proposed an infinite-dimensional H_∞_ control method to address feedback delays caused by high-resolution capacitive sensors in piezoelectric nano-positioning stages, where the experimental results outperformed traditional PID and finite-dimensional controllers. Makarem et al. [11] applied direct data-driven iterative tuning to optimize PID parameters for ultrasonic motors using dual-source and dual-frequency driving techniques. Wang et al. [12] introduced a predictive switching control scheme that could switch between two controllers based on predicted responses to reduce settling time and overshoots. A multiple-switching control framework proposed for PZT stages in [13,14] considered future responses by multiple control sequences to provide better performance than using a single-switching scheme. However, these response predictions were based on a nominal plant and ignored variations in operating conditions. For this reason, further system improvement will require updating the models in the response predictors.

Recent studies have highlighted the importance of accurate response prediction in control systems. For example, Smith et al. [15] presented a data-driven predictive control framework that leveraged past input–output trajectories to improve control performance by predicting future responses. Hiraoka et al. [16] developed a database-driven PID controller that could handle unknown time-delay systems through response prediction. This paper proposes a model estimation mechanism that can estimate the system model in real time and apply it to forecast responses, thereby improving both prediction accuracy and control performance. We can then integrate model estimation and control switching mechanisms and conduct simulations and experiments.

While the PZT stage can achieve nm-level precision, its travel range is limited. To overcome this limitation, we integrated it with a motor stage to provide long-range motion with nanometer-level precision. We then developed gain-scheduling and feedforward control frameworks for the motor stage. The former adjusts control gains based on tracking errors, while the latter compensates for phase lag, thereby improving overall system responsiveness and accuracy. Finally, we integrated the motor and PZT stages and verified the effects of the proposed control strategies through simulations and experiments. Subsequently, the integrated stage was employed to fabricate micro-lenses via two-photon polymerization. We then evaluated the lenses’ optical properties to highlight the merits of the proposed strategies.

The paper is arranged as follows: Section 2 depicts the precision long-travel stage and models. Section 3 introduces control switching and model estimation mechanisms for PZT stages. Section 4 depicts the gain-scheduling and feedforward mechanisms for motor stages. Section 5 integrates the stages and evaluates system performance through simulations and experiments. Section 6 applies the integrated stage to fabricate micro-lenses and evaluate the effectiveness of the control by characterizing the lenses’ optical properties. Finally, Section 7 concludes the study.

## 2. The Long-Travel Precision Stage

**Figure 1:**
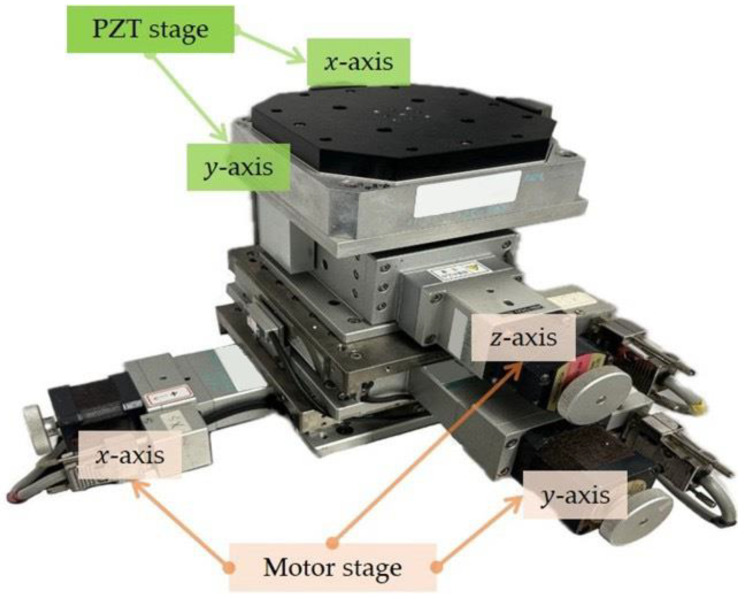
The precision long-travel stage.

This section depicts the long-travel precision system, which integrates PZT and motor stages (see Figure 1). The former has encoders with resolutions of 1.22 nm and a travel of 100 μm [17]. In contrast, the latter has a 100 mm-travel and 1 μm-resolution [18,19]. An interferometer was applied as a global sensor in [20] to verify the encoder-sensor layouts. The system specifications are available in Appendix A [17,18,19]. By combining these stages, the system can achieve nanometer-level positioning accuracy over centimeter-scale distances. This section also presents the modeling of both stages and the design of their respective control strategies.

### 2.1. PZT Stage Models

**Figure 2:**
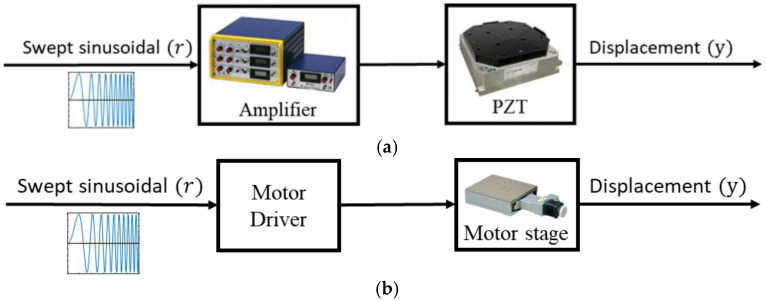
Derivation of stage models: (**a**) PZT stage; (**b**) motor stage.

We used experiments to derive the stage models, as shown in Figure 2a, by applying a swept sinusoidal signal as the system input *r* and measuring the stage displacements *y* to derive the stage model. An amplifier magnified the driving signals to regulate the stage travels within a range of 0–100 μm.

The identification processes were repeated to derive ten models for the x-axis, as illustrated in Appendix B. A nominal plant $G_{0}$ was then chosen for control designs in Section 3 based on gap metric principles [21]. Suppose $G_{0}={\tilde{M}}^{-1}\tilde{N}$, where $\tilde{M},\;\tilde{N}\in RH_{\infty }$ and $\tilde{M}{\tilde{M}}^{*}+\;\tilde{N}{\tilde{N}}^{*}=\;I.$ A perturbed plant $G_{\Delta }={\left(\tilde{M}+\Delta_{\tilde{M}}\right)}^{-1}\left(\tilde{N}+\Delta_{\tilde{N}}\right)$ represents the actual physical system and is different from the mathematical model $G_{0}$. The gap between $G_{0}$ and $G_{\Delta }$ is labeled as $\delta (G_{0},G_{\Delta })$, which is the smallest *ε* such that $\Delta =\left[\begin{array}{cc} \Delta_{\tilde{M}} & \Delta_{\tilde{N}} \end{array}\right]$ with ${\left\|\Delta \right\|}_{\infty }\leq \varepsilon$ can perturb $G_{0}$ into $G_{\Delta }$ [22]. Based on the gap analysis, we selected the following nominal plants for control designs:

(1) $$\begin{array}{l} G_{P}^{x}=\arg\underset{G_{i}^{x}}{\min}\underset{G_{j}^{x}}{\max}\delta_{g}(G_{i}^{x},G_{j}^{x}),\;i,j=1,\;2,\;\ldots ,\;10\\ =G_{3}^{x}=\frac{2.02\times 10^{3}s^{3}+1.22\times 10^{6}s^{2}+1.45\times 10^{9}s+3.25\times 10^{10}}{s^{4}+6.49\times 10^{2}s^{3}+1.02\times 10^{6}s^{2}+1.95\times 10^{8}s+3.60\times 10^{9}}, \end{array}$$

Here, $\delta (G_{P}^{x},G_{i}^{x})\leq 0.0556,\;\forall G_{i}^{x}$ minimizes the maximum gaps among all models.

Similarly, we derived ten models for the y-axis of the PZT stage, as illustrated in Appendix B, and selected the following nominal plant for control designs in Section 3:

(2) $$\begin{array}{l} G_{P}^{y}=\arg\underset{G_{i}^{y}}{\min}\underset{G_{j}^{y}}{\max}\delta_{g}(G_{i}^{y},G_{j}^{y}),\;i,j=1,\;2,\;\ldots ,\;10\\ =G_{5}^{y}=\frac{7.02\times 10^{2}s^{3}+4.25\times 10^{5}s^{2}+6.98\times 10^{8}s+3.64\times 10^{9}}{s^{4}+2.90\times 10^{2}s^{3}+5.07\times 10^{5}s^{2}+7.87\times 10^{7}s+2.02\times 10^{9}}, \end{array}$$

which minimizes the maximum gaps among all models, with $\delta (G_{P}^{y},G_{i}^{y})\leq 0.0810,\;\forall G_{i}^{y}$.

### 2.2. Motor Stage Models

The models of the motor stage were also derived through experiments by applying a signal *r* as input and measuring the output *y* (see Figure 2b). Based on gap analyses, the experiments were also repeated to derive ten models on each axis, as illustrated in Appendix B. Based on the gap metrics, the following nominal plants were then selected for control design in Section 4.

$$G_{M}^{x}=\frac{0.1007}{s},\;G_{M}^{y}=\frac{0.1009}{s}.$$

## 3. Control Switching and Model Estimation for the PZT Stage

**Figure 3:**
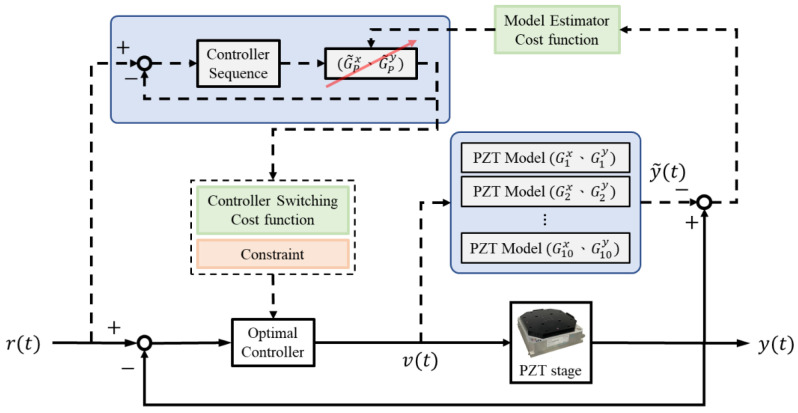
Model estimation and control switching for the PZT stage.

This section introduces the control switching and model estimation mechanisms for the PZT stage, as illustrated in Figure 3.

### 3.1. Robust Control Designs

We applied $G_{P}^{x}\;\mathrm{and}\;G_{P}^{y}$ for control designs for precision stages. The system’s internal stability can be maintained by a controller *K* for all plants $G_{\Delta }$ with $\delta (G_{0},G_{\Delta })\leq \varepsilon$ if the stability margin is as follows [23]:

$$b(G_{0},K)\equiv {\left\|\left[\begin{array}{l} K\\ I \end{array}\right]{\left(I-G_{0}K\right)}^{-1}[I\;G_{0}]\right\|}_{\infty }^{-1}>\varepsilon$$

**Figure 4:**
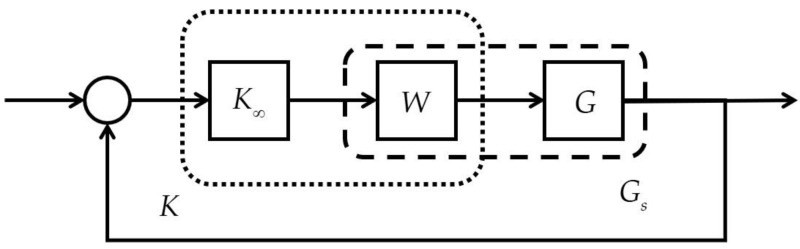
Loop shaping.

We designed controllers $K_{\infty }$ using the loop-shaping technique with a weighted plant $G_{s}=GW$, as illustrated in Figure 4. The selection of the weighting function *W* followed three key principles [24,25]: (1) increasing $\left|G_{s}(j\omega )\right|$ when ω is small; (2) decreasing $\left|G_{s}(j\omega )\right|$ when ω is large; and (3) letting the slope of $\left|dG_{s}(j\omega )/d\omega \right|\in \left[\begin{array}{cc} -40 & 0 \end{array}\right]\;\mathrm{dB}$ around the cross-over frequency. Finally, we implemented the weighted controller $K=WK_{\infty }$ to control G.

We applied the following weighting functions:

$$W_{f}^{x}=\frac{40(s+40\pi )}{s(s+30\pi )},\;W_{m}^{x}=\frac{20(s+15\pi )}{s(s+30\pi )}\;,\;W_{s}^{x}=\frac{15(s+10\pi )}{s(s+40\pi )}$$

$$W_{f}^{y}=\frac{40(s+60\pi )}{s(s+20\pi )},\;W_{m}^{y}=\frac{15(s+15\pi )}{s(s+40\pi )}\;,\;W_{s}^{y}=\frac{10(s+15\pi )}{s(s+40\pi )}$$

and designed the robust controllers using the MATLAB R2024a command *ncfsyn*:

$$\begin{array}{l} K_{f}^{x}=\frac{34.36s^{6}+2.83\times 10^{4}s^{5}+3.88\times 10^{10}s^{3}+1.29\times 10^{10}s^{2}+\;1.32\times 10^{14}s+5.02\times 10^{15}}{s^{7}+1.14\times 10^{3}s^{6}+1.31\times 10^{5}s^{5}+6.58\times 10^{8}s^{4}+9.62\times 10^{10}s^{3}+\;4.52\times 10^{12}s^{2}+6.03\times 10^{14}s},\\ K_{m}^{x}=\frac{30.80s^{6}+1.71\times 10^{4}s^{5}+1.65\times 10^{7}s^{4}+7.31\times 10^{9}s^{3}+7.40\times 10^{11}s^{2}+3.26\times 10^{13}s+1.75\times 10^{14}}{s^{7}+9.99\times 10^{2}s^{6}+8.71\times 10^{5}s^{5}+3.35\times 10^{8}s^{4}+4.28\times 10^{10}s^{3}+\;4.18\times 10^{12}s^{2}+9.88\times 10^{13}s},\\ K_{s}^{x}=\frac{11.10s^{6}+5.35\times 10^{3}s^{5}+6.77\times 10^{6}s^{4}+4.18\times 10^{9}s^{3}+5.32\times 10^{11}s^{2}+\;7.02\times 10^{13}s+1.05\times 10^{14}}{s^{7}+9.33\times 10^{2}s^{6}+7.29\times 10^{5}s^{5}+7.92\times 10^{8}s^{4}+3.88\times 10^{10}s^{3}+\;5.45\times 10^{12}s^{2}+9.87\times 10^{13}s}, \end{array}$$

$$\begin{array}{l} K_{f}^{y}=\frac{38.22s^{6}+5.38\times 10^{4}s^{5}+7.06\times 10^{7}s^{4}+2.22\times 10^{10}s^{3}+\;2.88\times 10^{12}s^{2}+1.58\times 10^{14}s+1.93\times 10^{15}}{s^{7}+1.01\times 10^{3}s^{6}+6.87\times 10^{5}s^{5}+5.25\times 10^{8}s^{4}+7.98\times 10^{10}s^{3}+4.76\times 10^{12}s^{2}+3.58\times 10^{13}s},\\ K_{m}^{y}=\frac{20.64s^{6}+8.78\times 10^{4}s^{5}+9.18\times 10^{6}s^{4}+5.38\times 10^{9}s^{3}+1.78\times 10^{11}s^{2}+8.98\times 10^{12}s+7.58\times 10^{13}}{s^{7}+7.14\times 10^{2}s^{6}+6.45\times 10^{5}s^{5}+3.61\times 10^{8}s^{4}+7.62\times 10^{10}s^{3}+1.55\times 10^{12}s^{2}+10.33\times 10^{13}s},\\ K_{s}^{y}=\frac{17.90s^{6}+6.38\times 10^{4}s^{5}+1.75\times 10^{7}s^{4}+5.69\times 10^{9}s^{3}+8.58\times 10^{11}s^{2}+3.22\times 10^{13}s+9.40\times 10^{14}}{s^{7}+9.03\times 10^{2}s^{6}+9.56\times 10^{5}s^{5}+5.01\times 10^{8}s^{4}+8.55\times 10^{10}s^{3}+5.37\times 10^{12}s^{2}+1.24\times 10^{13}s}. \end{array}$$

**Figure 5:**
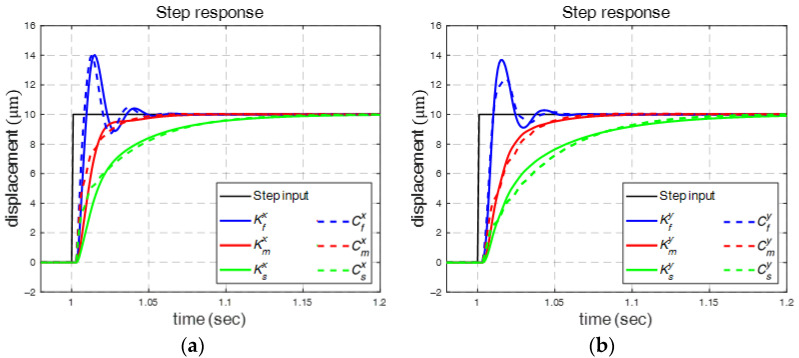
Response comparison with robust and robust PI controls: (**a**) *x*-axis; (**b**) *y*-axis.

$K_{f}^{i},\;K_{m}^{i},\;\mathrm{and}\;K_{s}^{i}$ denote the fast, medium, and smooth controllers, respectively, for the *i*-axis, where $i\in \left\{x,\;y\right\}$. These controllers represent different control characteristics, as shown in Figure 5, where $K_{f}^{x}\;\mathrm{and}\;K_{f}^{y}$ produce rapid yet oscillatory responses, while $K_{s}^{x}\;\mathrm{and}\;K_{s}^{y}$ provide smooth but slower responses, and $K_{m}^{x}\;\mathrm{and}\;K_{m}^{y}$ yield a compromise between the rapid and smooth controllers.

We then applied the PSO algorithms [26] to simplify the controllers $K_{f}^{x},\;K_{m}^{x},\;\mathrm{and}\;K_{s}^{x}$, as follows:

$$C_{f}^{x}(s)=0.088+\frac{28.330}{s},\;C_{m}^{x}(s)=0.099+\frac{7.387}{s},\;C_{s}^{x}(s)=0.083+\frac{4.645}{s}$$

$$C_{f}^{y}(s)=0.007+\frac{39.950}{s},\;C_{m}^{y}(s)=0.091+\frac{7.284}{s},\;C_{s}^{y}(s)=0.082+\frac{4.653}{s}$$

These controllers were then converted into discrete time for implementation. The system responses are compared in Figure 5, where $C_{f}^{x},\;C_{m}^{x},\;\mathrm{and}\;C_{s}^{x}$ provide similar responses as $K_{f}^{x},\;K_{m}^{x},\;\mathrm{and}\;K_{s}^{x}$ but with significantly simpler structures. Therefore, we applied these robust PI controllers to control the PZT stage.

### 3.2. Multiple-Switching Control

**Figure 6:**
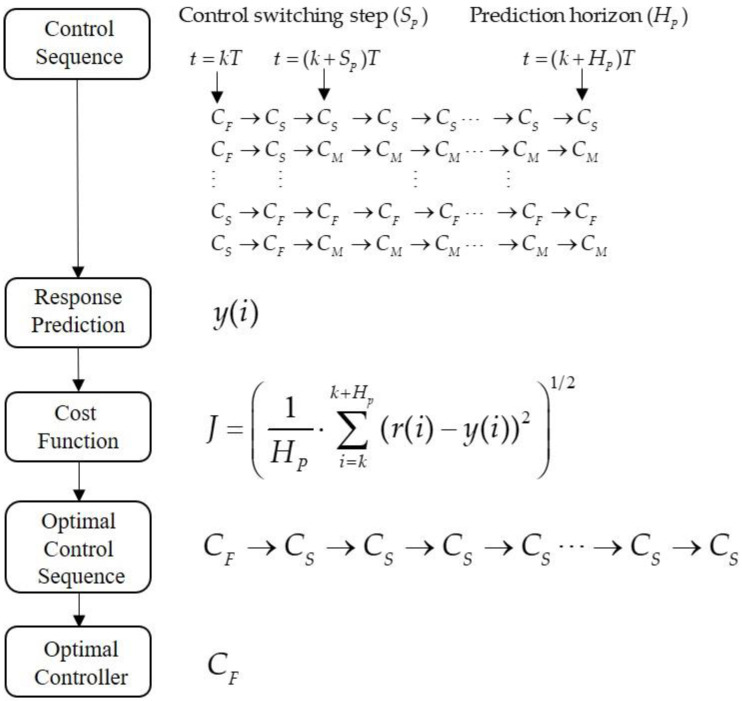
Multiple-switching control mechanism.

System control usually has multiple control objectives, such as fast responses and limited overshoots, which are difficult to achieve concurrently using a single controller. Therefore, we proposed a multiple-switching control mechanism to determine the most suitable control sequences by predicting system behaviors [14]. The conceptual flow of the integrated switching mechanism is illustrated in Figure 6. The response predictions are based on a controller switching mechanism with three components: (1) the prediction horizon ($H_{P}$), (2) the switching period ($S_{P}$), and (3) the cost function.

The response prediction compares the system responses in the future horizon $H_{P}$ using potential $3^{S_{P}}$ control sequences, as shown in Figure 6, and computes the root mean square error (RMSE)

(3) $$J={\left(\frac{1}{H_{P}}\cdot \sum_{i=k}^{k+H_{p}}{\left(r(i)-y(i)\right)}^{2}\right)}^{1/2}$$

as the cost function. The optimal control sequence, which achieves the minimum cost, is then selected. The corresponding controller is then applied to control the stage.

### 3.3. Model Estimation

Considering the model variation in the response predictor, we developed a model estimation mechanism that can update the stage model, $G_{i}^{x}$ and $G_{j}^{y}$, in real time, based on the model input v and output y when predicting the responses. The model was selected to minimize the estimation errors, as follows:

$${\tilde{G}}_{P}^{x}=\arg\underset{G_{i}^{x}}{\min}{\left(y(k)-\tilde{y}(k)\right)}^{2},\;{\tilde{G}}_{P}^{y}=\arg\underset{G_{i}^{y}}{\min}{\left(y(k)-\tilde{y}(k)\right)}^{2}$$

where y represents the measured output and $\tilde{y}$ is the estimated output when using $G_{i}^{x}\;\mathrm{and}\;G_{j}^{y},\;i,j=1,\;2,\ldots ,\;10$.

We further integrated the model estimation and multiple-switching control mechanisms to decide on the optimal control sequences in real time. Because the models are updated according to the model input v(t) and output y(t), we updated the corresponding controllers as follows:

$$\begin{array}{l} G_{i}^{x}C_{f,i}^{x}=G_{P}^{x}C_{f}^{x};\;G_{i}^{x}C_{m,i}^{x}=G_{P}^{x}C_{m}^{x};\;G_{i}^{x}C_{s,i}^{x}=G_{P}^{x}C_{s}^{x},\\ G_{i}^{y}C_{f,i}^{y}=G_{P}^{y}C_{f}^{y};\;G_{i}^{y}C_{m,i}^{y}=G_{P}^{y}C_{m}^{y};\;G_{i}^{y}C_{s,i}^{y}=G_{P}^{y}C_{s}^{y}, \end{array}$$

to maintain the same loop transfer functions. The modified controllers are summarized in Appendix C.

Implementing the control mechanisms, we conducted experiments with a step input of 10 μm. Table 1 shows the comparison of system performance, including the overshoot, RMSE, settling time, and rise time, to highlight the effects of the proposed mechanisms. Although the rise time shows a slight regression, its influence on overall performance does not pose a substantial issue and can be neglected.

## 4. Gain Scheduling and Feedforward for the Motor Stage

**Figure 7:**
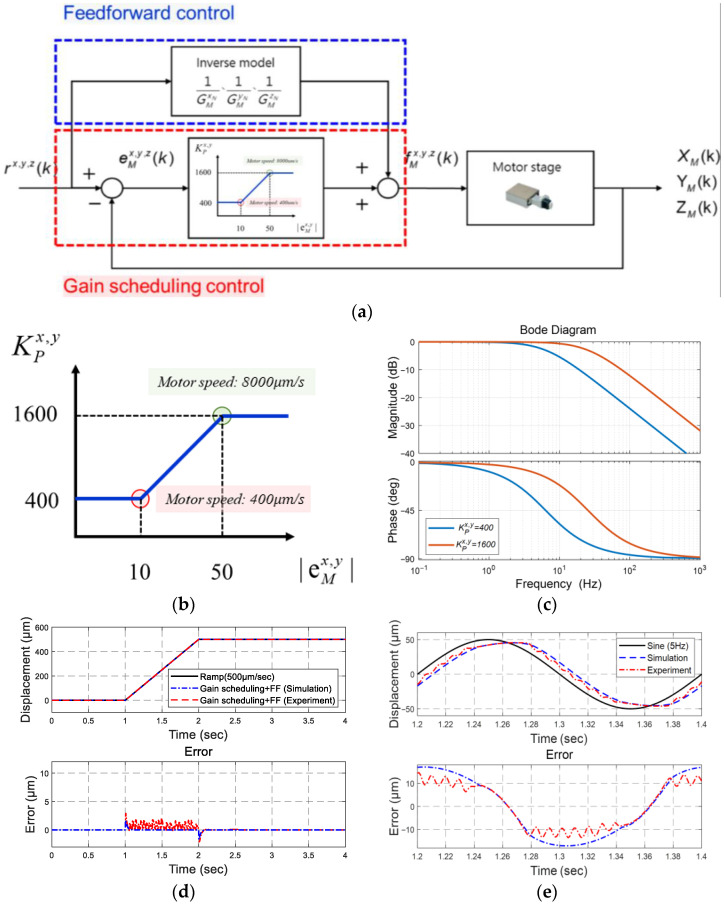
Motor stage control: (**a**) the block diagram; (**b**) gain scheduling; (**c**) Bode plots; (**d**) response to a ramp input; (**e**) response to a sinusoidal input.

We designed gain scheduling and feedforward mechanisms [13] for the motor stage (see Figure 7a). Because these are first-order models, a constant control $K_{p}$ can arbitrarily assign closed-loop poles. In addition, feedforward can enhance closed-loop responses by mitigating phase lags.

### 4.1. Gain Scheduling

We can adjust the controller gain $K_{p}$ accordingly to tracking errors $e_{M}$, as follows (see Figure 7b):

(4) $$\;K_{P}=\left\{\begin{array}{l} 1600,\;\mathrm{if}\;\left|e_{M}\right|>50\;\mu m,\\ 30\cdot \left(\left|e_{M}\right|-10\right)+400,\;\mathrm{if}\;10\leq \left|e_{M}\right|\leq 50\;\mu m\\ 400,\;if\;\left|e_{M}\right|<10\;\mu m. \end{array}\right.,$$

We set the maximum gain as 1600 because the maximum motor speed is 80,000 pulse/s, so the motor can reduce the tracking errors at the maximum speed when they are larger than 50 μm. The minimum gain was set to 400 when the tracking errors are less than 10 μm, allowing the PZT stage to compensate for the tracking errors of the combined stage smoothly.

The system’s Bode plots, illustrated in Figure 7c, show significant phase lags in the middle frequency range. Therefore, a feedforward mechanism was developed to reduce the phase lag.

### 4.2. Feedforward Compensation

A feedforward control can compensate for a phase lag by employing inverse motor-stage models. Since the inverse models are inherently improper, we designed the feedforward controllers by adding one additional pole, as follows:

$$C_{CFF}^{x}=\frac{s}{0.0001s+0.1007},\;C_{CFF}^{y}=\frac{s}{0.0001s+0.1009}$$

For example, given a ramp input of 500 μm/s and a sinusoidal input of 5 Hz, the tracking results shown in Figure 7d,e, and Table 2 illustrate the effects of the feedforward and gain scheduling control.

## 5. Integration of the Long-Travel Precision Stage

**Figure 8:**
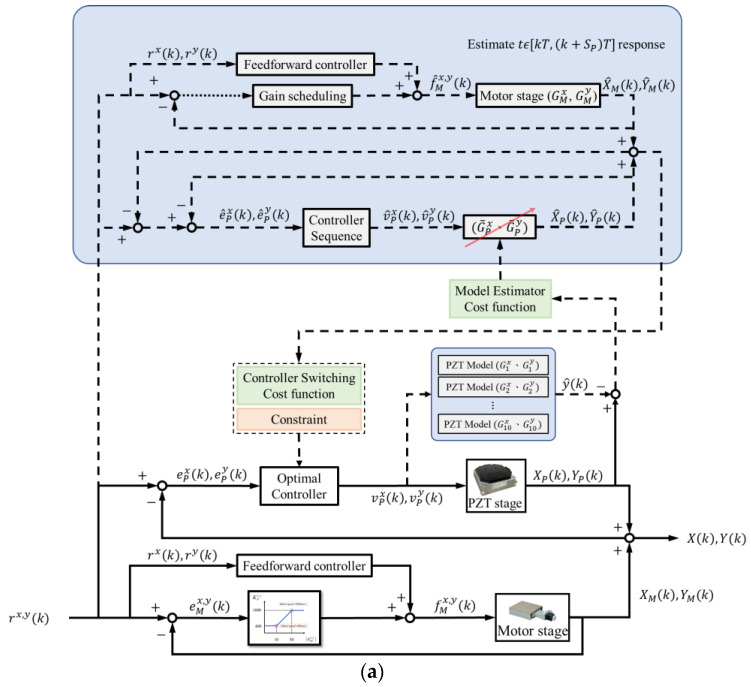

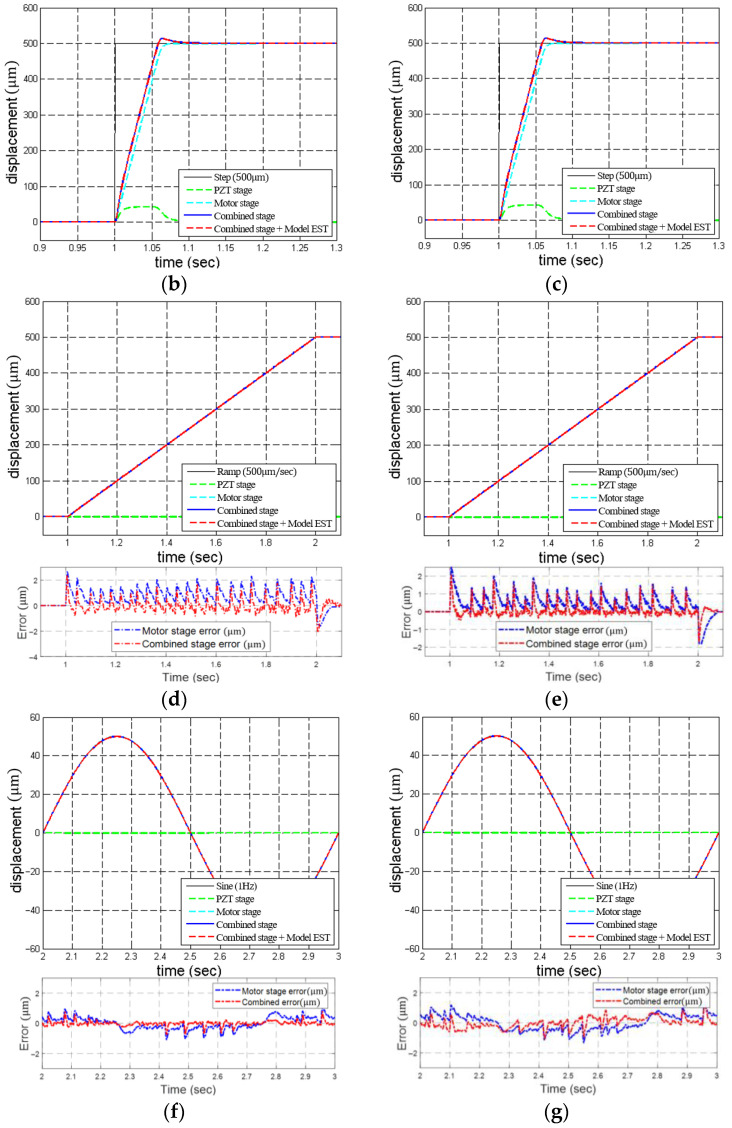
Combined stage control: (**a**) block diagram; (**b**) *x*-axial step response; (**c**) *y*-axial step response; (**d**) *x*-axial ramp response; (**e**) *y*-axial ramp response; (**f**) *x*-axial sinusoidal response; (**g**) *y*-axial sinusoidal response (*y*-axis).

The PZT and motor stages were integrated, as illustrated in Figure 8a. For the PZT stage, the model estimator selects the optimal model and updates the corresponding controllers, while the response predictor forecasts the system responses and chooses the best control sequences. For the motor stage, the control gains were modified according to the tracking errors, while the feedforward control compensated for the phase lags.

Because the PZT had limited travel, the following function was implemented to prevent saturation:

(5) $$e_{P}^{i}(k)=\left\{\begin{array}{l} 0,\;\mathrm{if}\;\left|e(k)\right|\geq 50\;\mu m\\ e(k),\;\mathrm{otherwise} \end{array}\right.,\;i\in \left\{x,\;y\right\}$$

where $e^{x}(k)=r^{x}(k)-X_{P}(k)-X_{M}(k)$ and $e^{y}(k)=r^{y}(k)-Y_{P}(k)-Y_{M}(k)$ denote the tracking errors of the integrated stage. The motor stage was applied for large-range positioning. Once the overall tracking error was less than 50 μm, the PZT stage could then achieve precision positioning by compensating for the errors $e_{P}^{x}\;\mathrm{and}\;e_{P}^{y}$.

We implemented control mechanisms and conducted experiments with the following inputs: 500 μm step, 500 μm/s ramp, and 1 Hz sinusoidal signals. The responses are shown in Figure 8 and Table 3. Compared to the motor stage alone, the integrated stage with the model estimation and control switching mechanisms significantly reduced the overshoot and maximum errors. Moreover, a notable reduction in RMSE was also achieved. For example, compared with the multiple-switching control developed in [13], the proposed multiple control mechanisms improve the RMSE from 150.2 μm to 73.40 μm in the 500 μm step responses. Although the motor stage was equipped with a feedforward controller, noticeable tracking errors occurred during high-speed sinusoidal motion when using the motor stage alone. By incorporating the PZT stage for real-time error compensation, the integrated system was able to reduce tracking errors further. For instance, the PZT stages improved the tracking errors from 41% to 95%.

## 6. Micro-Lens Fabrication

**Figure 9:**
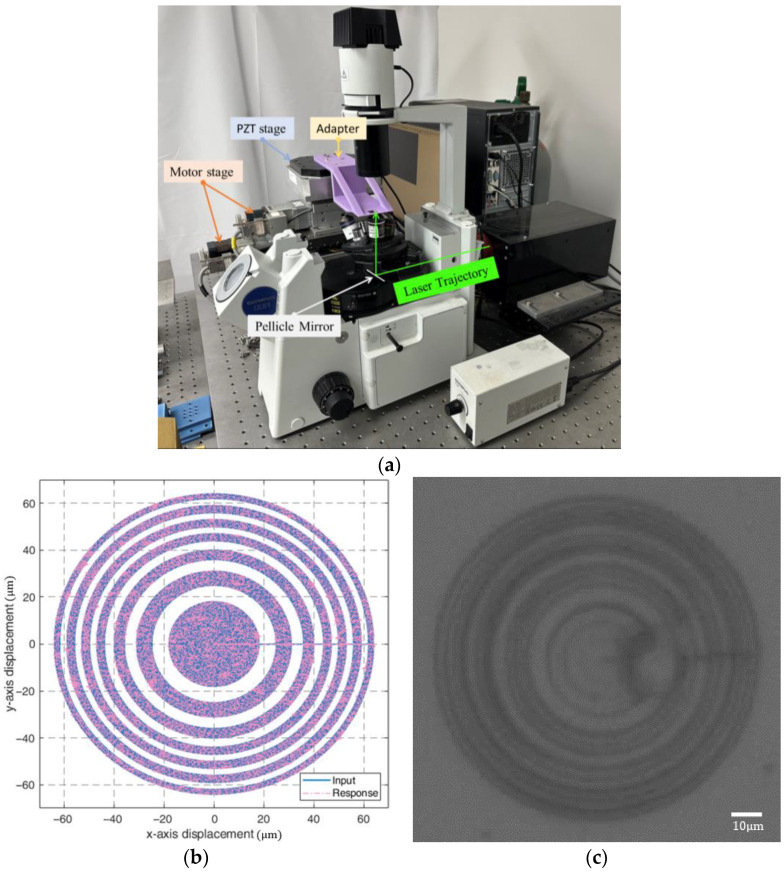
Micro-lens fabrication: (**a**) system integration; (**b**) FZP design; and (**c**) the micro-lens.

This section applies the combined stage to manufacture micro-lenses using a TPP system, which applies two-photon absorption (TPA) techniques and has been widely applied in micro-nano photonics [27,28] and micro-electromechanical systems [29,30]. The TPP system was integrated with the long-travel precision stage, as illustrated in Figure 9a, to fabricate micro-lenses. The mirror reflected the laser to focus into the resin on a microscope slide on the adapter attached to the PZT stage to manufacture micro-lenses by hardening the resin. The specially formulated resin is a blend of OrmoComp and photo-initiator for polymerization.

We fabricated a Fresnel Zone Plate (FZP), which focuses light using a planar micro-lens, with a diameter of 106 μm, with the following [14]:

(6) $$r_{n}={\left(nf\lambda +\frac{1}{4}n^{2}\lambda^{2}\right)}^{1/2}$$

as shown in Figure 9b, where $r_{n}$ is the radius of circle *n*, *f* is the focal length, while λ is the wavelength. We set the number of zones as *n* = 9, *f* = 500 μm, and λ=632.8 nm. The fabricated micro-lens is shown in Figure 9c.

**Figure 10:**
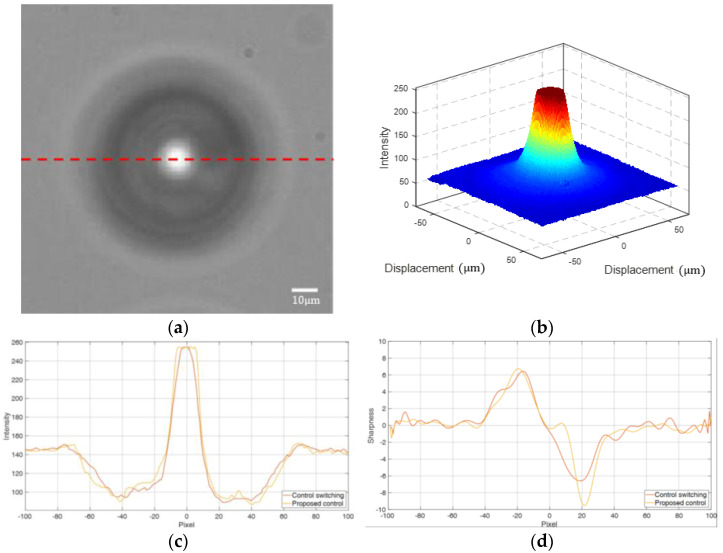
FZP: (**a**) focal plane; (**b**) image brightness; (**c**) the intensity curves; and (**d**) the sharpness curves.

We used each micro-lens to focus light on the focal plane of a camera and assessed its optical quality based on the image intensity and sharpness, as illustrated in Figure 10a. Each captured image was converted to grayscale, where the brightness of each pixel was labeled between 0 and 255, where 0 represents completely black and 255 represents completely white, as shown in Figure 10b. The brightness on the dashed line is shown in Figure 10c, where intensity is defined as the corresponding grayscale value on each pixel, while sharpness is the derivative of the intensity (see Figure 10d).

The stage’s tracking errors and the micro-lenses’ optical properties are illustrated in Table 4. Compared with the micro-lenses fabricated in [31], which had a maximum intensity of 160, the multiple-switching mechanism in [14] improved the maximum intensity to 255 with a maximum sharpness of 6.6. In addition, the proposed multiple control mechanisms slightly reduced the RMSE from 94.2 nm to 92.5 nm and significantly increased the maximum sharpness to 9.5. These results confirm the merits of the multiple control mechanisms for enhancing the microfabrication precision.

## 7. Conclusions

In this study, we developed multiple control mechanisms for a precision long-travel stage consisting of a PZT and motor stages. The former used the control switching and model estimation mechanisms, while the latter utilized the gain scheduling and feedforward controls. Ultimately, the integrated stage enabled long travel with precision positioning. We implemented this multiple control strategy in a combined stage and demonstrated its effectiveness in enhancing precision positioning in simulations and experiments. Finally, we applied the long-travel precision stage to fabricate micro-lenses using TPP and demonstrated the advantages of the control mechanisms in microfabrication processes by evaluating the lenses’ optical characteristics.

## Figures and Tables

**Table 1:** Tracking performance of the PZT stage (step responses).

			C.S. [14]	Proposed Control	Imp. (%)
*x*-axis	Simulation	Overshoot (%)	3.9786	1.8051	54.63
		RMSE (μm)	1.6949	1.5126	1.08
		Settling time (s)	1.0291	1.0114	1.72
		Rising time (s)	0.0061	0.0064	−4.92
	Experiment	Overshoot (%)	8.9100	3.9200	56.00
		RMSE (μm)	1.4550	1.4092	3.15
		Settling time (s)	1.0712	1.0503	1.96
		Rising time (s)	0.0066	0.0069	−4.55
*y*-axis	Simulation	Overshoot (%)	3.7525	1.7585	53.14
		RMSE (μm)	1.4816	1.4681	0.91
		Settling time (s)	1.0789	1.0117	6.23
		Rising time (s)	0.0060	0.0065	−8.33
	Experiment	Overshoot (%)	2.2522	1.5104	32.90
		RMSE (μm)	1.4848	1.4672	1.20
		Settling time (s)	1.0207	1.0115	0.90
		Rising time (s)	0.0064	0.0066	−3.13

C.S.: control switching; imp. (%): percentage improvement.

**Table 2:** Performance: motor stage.

		Ramp Input (500 μm/s)	Sinusoidal Input (5 Hz)
Simulation	Phase lag (deg)	-	2.4000
	MAE (μm)	1.6618	3.6400
	RMSE (μm)	0.1998	2.5352
Experiment	Phase lag (deg)	-	3.6000
	MAE (μm)	3.0000	4.7922
	RMSE (μm)	0.9122	4.1897

MAE: maximum absolute error.

**Table 3:** Experimental data analysis.

		*x*-Axis		*y*-Axis	
		Motor Stage	Combined Stage	Motor Stage	Combined Stage
Step	Rising time (s)	0.0487	0.0452	0.0501	0.0487
	Settling time (s)	1.0674	1.0639	1.0852	1.0831
	Overshoot (%)	0	1.5200	0	1.4191
	RMSE (μm)	76.3951	76.3822	87.4249	87.4209
Ramp	MAE (μm)	2.5000	2.4000	2.9000	2.9000
	RMSE (μm)	0.7851	0.3958	0.9122	0.5301
Sinusoidal	Gain (dB)	0.2229	0.0115	0.3072	0.0454
	MAE (μm)	3.6000	0.7849	3.0000	0.9755
	Phase lag (μm)	4.7922	1.4390	5.5460	1.4162
	RMSE (μm)	4.1897	0.1687	4.0452	0.3321

MAE: maximum absolute error.

**Table 4:** FZP performance.

	C.S. [14]	Proposed Control
RMSE (nm)	94.2	92.5
Maximum Intensity	255	255
Maximum Sharpness	6.6	9.5

C.S.: control switching.

## Data Availability

The original contributions presented in this study are included in the article. Further inquiries can be directed to the corresponding author.

## Appendix A. Stage Specifications

The system specifications are available at https://reurl.cc/mYxOOV (accessed on 29 August 2025).

## Appendix B. Stage Models

The system models are available at https://reurl.cc/NxYM75 (accessed on 29 August 2025).

## Appendix C. Modified Controllers

The modified controllers are available at https://reurl.cc/NxYMvq (accessed on 29 August 2025).
